# The value of cell-free circulating tumour DNA profiling in advanced non-small cell lung cancer (NSCLC) management

**DOI:** 10.1186/s12935-021-02382-0

**Published:** 2021-12-16

**Authors:** Maria Gabriela O. Fernandes, Natália Cruz-Martins, José Carlos Machado, José Luís Costa, Venceslau Hespanhol

**Affiliations:** 1 Pulmonology Department, Centro Hospitalar Universitário de São João, Alameda Prof. Hernâni Monteiro, 4200-319 Porto, Portugal; 2 Faculty of Medicine, University of Porto, Alameda Prof. Hernani Monteiro, 4200-319 Porto, Portugal; 3 Institute of Molecular Pathology and Immunology of the University of Porto (IPATIMUP), 4200-135 Porto, Portugal; 4 Institute for Research and Innovation in Health (i3S), University of Porto, Rua Alfredo Allen, 4200-135 Porto, Portugal

**Keywords:** Lung cancer, Adenocarcinoma, Tumour genotyping, Liquid biopsy, Circulating cell-free tumour DNA, Next Generation Sequencing

## Abstract

Liquid biopsy (LB) has boosted a remarkable change in the management of cancer patients by contributing to tumour genomic profiling. Plasma circulating cell-free tumour DNA (ctDNA) is the most widely searched tumour-related element for clinical application. Specifically, for patients with lung cancer, LB has revealed valuable to detect the diversity of targetable genomic alterations and to detect and monitor the emergence of resistance mechanisms. Furthermore, its non-invasive nature helps to overcome the difficulty in obtaining tissue samples, offering a comprehensive view about tumour diversity. However, the use of the LB to support diagnostic and therapeutic decisions still needs further clarification. In this sense, this review aims to provide a critical view of the clinical importance of plasma ctDNA analysis, the most widely applied LB, and its limitations while anticipating concepts that will intersect the present and future of LB in non-small cell lung cancer patients.

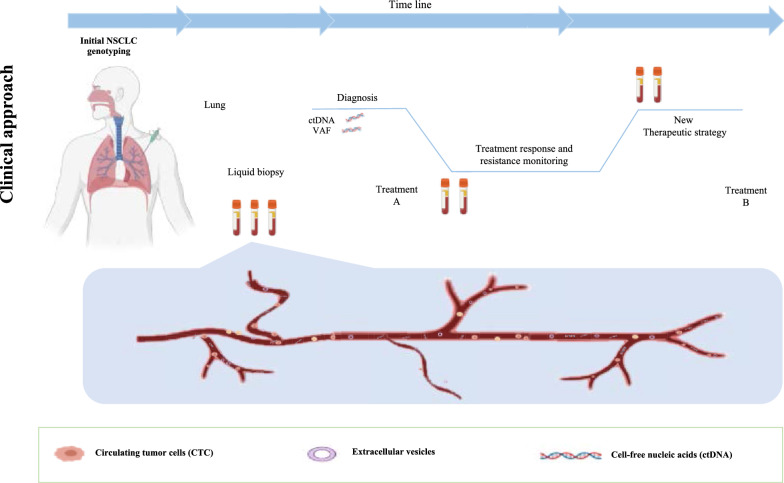

## Introduction

Lung cancer (LC) is the second most prevalent cancer globally, corresponding to 11.4% of diagnosed cancers, according to GLOBOCAN 2020 estimates, and it is the first cause of cancer death, accounting for 18% of deaths [1]. In the majority of cases, it presents with advanced or metastatic disease [2].

Numerous actionable genomic alterations have been identified in patients with advanced non-small cell lung cancer (NSCLC), particularly those with the adenocarcinoma subtype. As a result, targeted therapies have emerged, and LC treatment has become biomarker-driven [3, 4]. There are targeted treatments for genomic alterations in the *ALK, BRAF, EGFR, ERBB2, KRAS, MET, NTRK, RET and ROS1* genes, configuring substantial improvements in patient's survival and quality of life [3, 4]. The first targeted drug was approved for patients with *EGFR* activating mutations occurring in the tyrosine kinase domain of the gene, present in 15–20% of Caucasian patients with lung adenocarcinoma and in 40% of Asians [5–7]. Deletions of exon 19 (del 19) and the substitution of the amino acid p.Leu858Arg in exon 21 (L785R) comprise about 80–90% of the mutation spectrum. Rarer variants can also occur in exons 18 and 20, but their association with treatment response is less consistent [5]. Presently, for treating patients with tumours harbouring activating mutations in the *EGFR* gene 1st, 2nd and 3rd generation TKIs are approved, differing from each other on the receptor affinity and selectivity to different variants and providing a median progression-free survival (PFS) of 10 to 19 months [8–11]. In about 50–60% of patients treated with 1st or 2nd generation TKIs, the acquired resistance mechanism is a p.Thr790Met point mutation (T790M) in the *EGFR* gene [12–14]. This mutation increases the receptor affinity for ATP binding, drastically reducing the drug activity [15]. Third-generation TKIs have emerged as selective for both *EGFR* activating and T790M resistance mutations [16] with superior activity than chemotherapy in patients whose disease progressed with the T790M [17]. Still, disease progression-associated mechanisms are heterogeneous and not fully understood, differing whether the 3rd generation TKI is used at the frontline or after progression on 1st or 2nd generation TKIs [18–20].

With different proportions, *EGFR*-dependent mechanisms include new tertiary mutations, such as the exon 20 C797S mutation, *EGFR* amplification or T790M disappearance. The *EGFR* independent mechanisms can occur with bypass pathway activation, such as *ERB-B2* receptor tyrosine kinase 2 (*HER2*) and *MET* amplification, *PIK3CA* activating mutations, phosphatase and tensin homolog (*PTEN*) deletion, *RAS* mutations, and fusions affecting anaplastic lymphoma kinase (*ALK*) and ret-proto-oncogene (*RET*). Moreover, there is also the possibility of phenotypic alteration, such as small-cell lung cancer (SCLC) transformation [18–20].

*ALK* rearrangements occur in 3–5% of lung adenocarcinomas [21], consequent to an inversion on the short-arm of chromosome 2 joining its 3′ end with the 5′ end of the echinoderm microtubule-associated protein-like 4 (*EML4*), resulting in an *EML4*-*ALK* chimeric protein [22]. Targeted therapy for patients with *ALK* rearrangements have significantly impacted prognosis, with patients treated with a sequence of TKIs achieving over five years of survival after diagnosis of metastatic disease [23]. Tyrosine kinase inhibitors of different generations were developed, with relevant differences concerning *ALK* inhibitory potency, intracranial activity and efficacy on *ALK* mutations associated with resistance [24]. For patients with *ALK* fusions treated with TKIs, mutations in the *ALK* gene are one of the on-target resistance mechanisms. However, unlike *EGFR*, mutations are diverse and differ depending on each *ALK* inhibitor [25]. In addition, non-targeted alterations can occur alongside mutations and amplifications in different genes. For example, *MET* amplifications are present in about 15% of patients treated with new-generation TKIs, and histological transformation and epithelial-mesenchymal transition (*EMT*) may also occur [26, 27]. Therefore, recognising genomic alterations is essential for the selection and sequencing of *ALK* inhibitors.

Similar good results have been obtained with TKIs for other molecular targets. For example, synergising a *BRAF* inhibitor and a MEK inhibitor is indicated for NSCLC with *BRAF* p.V600E [3, 4]. For ROS proto-oncogene 1 (*ROS1*), *RET* and neurotrophic receptor tyrosine kinase 1 *(NTRK*) fusions, highly effective TKIs are available, as for the *MET* exon14 skipping mutation [3, 4]. For *ERBB2* mutations, TKIs and new antibody conjugates are being investigated [3]. The most recent advance in targeted therapy comprises the inhibition of the p.Gly12Cys mutation in the *KRAS* gene, one of the most frequent events in lung adenocarcinoma [3, 28].

In this sense, tumour genotyping is currently a fundamental element to determine the optimal treatment for each patient. Molecular tests are advised for untreated non-squamous NSCLC patients with advanced disease and others with clinical features linked to a greater probability of having driver mutations [4, 29]. Nonetheless, and despite is currently considered the gold standard, tissue biopsy is associated with numerous drawbacks. Tumour samples refer to small biopsies and cytology specimens obtained by invasive methods, as bronchoscopy, transthoracic biopsies, and pleural techniques. Also, not all tumour lesions are accessible, and tissue genotyping is linked to a 5–10% failure rate due to inadequate or insufficient DNA content [30]. In addition, tissue biopsies may not fully reflect tumour heterogeneity, as they are usually obtained from the most accessible tumour location site [31].

Over time, clinicians and researchers have pursued the idea of using non-invasive techniques for tumour diagnosis through a deeper study of peripheral blood and other fluids. Indeed, tumours release part of themselves into the circulation through the form of free nucleic acids, tumour cells, exosomes, among other elements, that can be extracted and analysed [32]. Fortunately, the advances stated in sequencing technologies have been a determinant step in making this ambition a reality.

Liquid biopsy (LB) is a non-invasive, easily taken, repeatable and less expensive technique than tissue biopsy and potentially reflects the heterogeneity of the genomic landscape, as it gets biological information from all tumour shedding sources [32]. A LB consists of analysing tumour-related biomarkers in body fluids, like blood, cerebrospinal fluid, pleural, pericardial effusions, and urine. It is a source of circulating cell-free DNA (cfDNA), circulating cell-free tumour DNA (ctDNA), circulating tumour cells (CTCs), exosomes, microRNAs, as well as proteins derived from cancer cells [32]. These components have distinct properties, potentialities and methods of capture and analysis, requiring further validation for clinical use, as briefly exposed in Table 1 [32–36].

**Table 1:** Tumour-related components characteristics and potentialities

Components	Characteristics and potential applications
CTC	Cells found in the blood of patients with solid tumours Surrogate marker for tumour growth and aggressiveness Genomic analysis
Exosomes	Cell-derived extracellular vesicles containing proteins, DNA, mRNAs, and miRNAs Biomarker analysis Potential therapeutic application
Circulating RNAs	miRNAs are the most abundant circulating RNA molecules miRNAs amount and composition differ between cancer and non-cancer landscape and correlates with that of solid tumours Potential early diagnosis biomarker
ctDNA	Tumour-derived fragmented DNA in the bloodstream that is not associated with cells Detection of oncogenic mutations, prognostic biomarker, tumour burden and minimal residual detection ctDNA methylation profiles
Tumour educated Platelets (TEPs)	TEPs may offer certain advantages over other blood-based biosources, including their abundance and easy isolation, high-quality RNA, and capacity to process RNA in response to external signals Different RNA profiles in platelets from cancer patients and healthy individuals
Proteins	Protein/proteome profile as a biomarker for cancer detection; predictive and prognostic biomarkers

*CTC* circulating tumour cell, *ctDNA* circulating tumour DNA, *mRNA* messenger RNA, *miRNAS* microRNAs

Cell-free DNA has been the most studied component, with ctDNA being the portion of cfDNA delivered by the tumour [37]. In these DNA fractions released by tumour apoptosis and necrosis and some active secretion [38], mutations in cancer-associated genes, microsatellite instability, and epigenetic alterations have been identified [39]. Cell-free circulating tumour DNA represents less than 1% of cfDNA [40], requiring highly sensitive analytic methods [41, 42]. Different sequencing technologies have been developed to detect mutant DNA and have evolved to achieve higher performance. They can be broadly grouped into two approaches: digital PCR and NGS-based methods [39, 43]. Both approaches have strengths and limitations. PCR-based assays are highly sensitive, able to detect variants with a frequency as low as 0.01%, less expensive and straightforward than NGS, but restricted to the detection of limited pre-planned alterations [40, 44]. NGS approaches are more complex but allow the detection of multiple alterations in different genes simultaneously. They can embrace "whole" alterations or be selected for targeted panels, being this one the most used for clinical application due to the highest sensitivity, lower cost, and simplicity of interpretation. Generally, NGS techniques can be amplicon-based or hybrid-capture-based, accounting for differences in test performance and the range of alterations capable of being detected [39, 44].

In the NSCLC setting, LB, particularly ctDNA genomic analysis, has an expanding role in detecting oncogenic driver alterations as well as emerging resistance mechanisms [45]. Thus, considering the role of LB in the most relevant clinical scenarios, we aim to provide a critical view of its importance and limitations while anticipating concepts intersecting the present and future clinical uses of LB in NSCLC patients, considering our "real-world" experience towards LB implementation [46, 47]. Specifically, in this review, we will discuss the application of LB for genotyping LC in its most relevant scenarios, for detection of resistance-related mutations, disease monitoring, with a focus being also given to the future applications of LB, reflecting on results’ interpretation and pitfalls.

## Circulating cell-free tumour DNA for detection of EGFR mutations

The detection of *EGFR* mutations, either activating or resistance-associated, is extremely relevant, given the link between *EGFR* mutations, treatment response and clinical outcomes. However, genetic testing for detection of *EGFR* mutations is not always successful, and re-biopsies display numerous difficulties, as previously mentioned. Before ctDNA genotyping is accepted as a surrogate for tissue genotyping, it is essential to reflect on the test accuracy and its predictive value as a biomarker for treatment selection. Several studies have addressed the analytical agreement between the mutational status assessed in plasma and tumour samples, and, in general, a robust correlation was found (Table 2). The meta-analysis (Table 3) published so far have demonstrated a sensitivity for detecting *EGFR* mutations ranging from 60 to 70% and a specificity of 80–98% [48–53]. Distinct studies with different technologies were included, that ultimately accounted for highly variable sensitivity values. For instance, when the effectiveness for detecting *EGFR* mutations with LB was addressed in a "real-world" setting, as in the multicentric studies, IGNITE and ASSESS [54, 55] (Table 2), plasma sensitivity was below 50%, with significant variability between centres and the technique used. Such findings reinforce the need to standardise procedures and validate techniques for large-scale implementation. The latest ultrasensitive sequencing technologies, such as digital PCR or plasma digital droplet PCR (ddPCR), use probes that allow the detection of del19 and L858R with very high sensitivity rates (greater than 80%) and specificity of 100% [56, 57] (Table 2). Moreover, it has become possible to analyse cfDNA by NGS, with advantages in sensitivity and wealth of information. Either amplicon-based [58, 59] or hybrid-capture sequencing [60, 61] have shown sensitivity reaching 94% and specificity exceeding 95% (Table 4).

**Table 2:** ctDNA plasma detection of EGFR mutation

Reference	Method of detection	Study type/ Sample size	Sensitivity/ PPA	Specificity/NPA	Concordance/ OPA between tissue and liquid biopsy
Douillard J et al. [65]	QUIAGEN therascreen® EGFR RGQ PCR Kit	Prospective, single-arm phase IV study (IFUM study); N = 652	65.7%	99.8%	94.3%
Reck M et al. [54]	QUIAGEN therascreen® EGFR RGQ PCR Kit; Cobas® EGFR mutations test version 2; Cycleave®; PNA-LNA PCR Clamp; other	Multicenter (ASSESS study); N = 1162	46%	97%	89%
Han B et al. [55]	Cobas® EGFR mutations test version 2	Multicenter (IGNITE study); N = 2561	46.9%	95.6%	80.5%
Wu YL et al. [148]	QUIAGEN therascreen® EGFR RGQ PCR Kit	Phase III, Prospective (Lux-Lung 3 and 6 studies); N = 334 (plasma); N = 287 (serum)	60.5% (plasma) 28.6% (serum)	n.r	n.r
Jenkins et al. [74]	Cobas® EGFR Mutation Test v2	AURA extension and AURA2 phase II studies; N = 210	T790M: 61% L858R: 76% Del19: 91%	T790M: 79% L858R: 98% Del19: 98%	T790M: 65% L858R: 85% Del19: 90%
Oxnard et al. [76]	BEAMing (Sysmex®)	Retrospective (AURA phase I) N = 216	T790M: 70% L858R: 86% Del19: 82%	T790M: 69% L858R: 97% Del19: 98%	n.r
Karlovich et al. [73]	Cobas® EGFR mutations test; BEAMing (Symex® Inostics GmbH)	Prospective, multicentre, observational and phase-1 TIGER-X; N = 153	(Cobas/BEAMING) Activating mutations: 73%/82% T790M: 64%/73%	(Cobas/BEAMING) Activating mutations: 100% T790M: 98%/50%	(Cobas/BEAMING) Activating mutations: 80% T790M: 86%/67%
Sacher et al. [56]	Droplet digital PCR (ddPCR)	Prospective; N = 180 (120 newly diagnosed + 60 acquired resistance)	Del19: 82% L858R: 74% T790M: 77%	Del19:100% L858R: 100% T790M: 63%	Del19: 91% L858R: 80% T790M: 40%
Zheng D et al. [57]	Droplet digital PCR (ddPCR)	N = 117	T790M: 81%	T790M: 100%	88%

*PPA* Positive percent agreement, *NPA* Negative percent agreement, *OPA* overall percent agreement, *n.r.* not reported

**Table 3:** Meta-analysis on the diagnostic accuracy of LB for detecting *EGFR* mutations

Reference	Study type/ Sample size	Sensitivity/PPA	Specificity/NPA	Concordance/ OPA between tissue and liquid biopsy
Quian et al. [48]	27 studies N = 3938	60%	94%	n.r
Luo et al. [49]	20 studies N = 2012	67.4%	93.5%	n.r
Qiu M et al. [50]	27 studies N = 3110	62%	95.9%	n.r
Mao et al. [51]	25 studies N = 2605	61%	90%	79%
Zhou et al. [52]	32 studies N = 4527	70%	98%	n.r
Passiglia et al. [53]	21 studies N = 1639	67%	80%	n.r

*PPA* Positive percent agreement, *NPA* Negative percent agreement, *OPA* overall percent agreement, *n.r.* not reported

**Table 4:** NGS-based studies analysing *cf*DNA for EGFR mutation detection

Reference	Method of detection	Study type/ Sample size	Sensitivity/ PPA	Specificity/ NPA	Concordance/ OPA between tissue and liquid biopsy
Kukita Y et al. [58]	NGS amplicon-based (Ion Torrent PGM®)	Retrospective n = 155 (144 plasma and 11 other fluids)	Del 19: 73% L858R or L861Q: 78%	n.r	n.r
		Prospective n = 22	78%	92%	86%
Reckamp et al. [59]	NGS Amplicon -based (Illumina MiSeq platform®)	Retrospective (TIGER-X study) N = 60 (urine and plasma)	T790M 93% L858R 100% Del19 87% (urine: T790M 72%; L858R 75% Del19 67%)	T790M 94% L858R 100% Del19 96% (urine: T790M 96%; L858R 100% Del19 94%)	n.r
Papadimitrakopoulou V et al. [60]	Cobas® EGFR Mutation Test v2;	Retrospective analysis from AURA 3 study N = 562	T790M: 51% L858R: 68% Del19: 82%	T790M: 77% L858R: 99% Del 19: 99%	T790M: 61% L858R: 88% Del 19: 89%
	ddPCR (Biodesix®)		T790M: 58% L858R: 70% Del19: 73%	T790M: NA L858R: 98% Del 19: 100%	n.r
	NGS (Guardant360®, Guardant Health)		T790M: 66% L858R: 63% Del19: 79%	T790M: NA L858R: 98% Del 19: 99%	n.r
Schwartzberg et al. [61]	NGS HiSeq® 2500 (Illumina)	Prospective N = 117	94%	100%	94%

*PPA* Positive percent agreement, *NPA* Negative percent agreement, *OPA* overall percent agreement. *n.r.* not reported

The first data considering the predictive value of cfDNA for response to *EGFR* TKIs came from the trial comparing chemotherapy with a 1st generation TKI [62, 63]. Goto et al. [63] firstly found a significant correlation between cfDNA *EGFR* mutation status and PFS, and although the serum test had low sensitivity (43.1%), it opened the window for further investigation. The clinical utility of plasma *EGFR* mutation detection and the concordance between the mutational status in plasma and tissue were investigated in *EGFR*-mutated patients undergoing 1st line treatment with TKIs, with sensitivity, specificity, and concordance rates of 66.5, 99.8 and 94.3%, respectively [64]. Also, OS and PFS did not differ regardless of whether the mutation was detected in plasma or tissue [64, 65]. Besides that, plasma allowed the detection of additional cases that were not identified in the available tissue sample [64]. Likewise, in a retrospective analysis of a 1st generation TKI *versus* standard chemotherapy as 1st line treatment for European patients with advanced *EGFR* mutation-positive NSCLC, the plasma detection of *EGFR* mutations by real-time PCR showed a predictive capacity with an OS and PFS overlapping that of tissue [66]. These data were of enough robustness, demonstrating a strong association between detection of plasma mutations and response to TKIs, leading to the first approval of a LB for detecting *EGFR* mutations [67] (Table 5).

**Table 5:** Characteristics of commercially approved platforms for ctDNA

Methodology	Assay	Technique	Sample	Gene Spectrum	Approved indications
Allelic-specific PCR	Cobas EGFR mutation Test v2®	Real-time PCR	DNA derived from FFPE tissue or cfDNA from plasma	42 EGFR mutations in exons 18, 19, 20, 21	FDA approval for detection of EGFR del19, EGFR L858R, and EGFR T790M; FDA, Jun and Sept, 2016 [67]
	Therascreen® EGFR Plasma RGQ PCR kit	Real-time PCR	cfDNA from plasma	29 EGFR mutations in exons 19, 20, 21	E.U. approval for detection of EGFR del19 and EGFR L858R; EMA, Jan 2015 [78]
	AmoyDx Super-ARMS® EFGR mutation test kit	Real-time PCR	Compatible with FFPE tissue or plasma/serum samples	41 EGFR mutations in exons 18–21, including L858R, exon 19 deletions, and T790M	Chinese FDA approval for detection of EGFR del19, EGFR 858R, and EGFR T790M China FDA. Jan 2018 [79]
NGS	Guardant360 CDx®	Targeted hybridization-based capture technology	cfDNA	73-gene panel (single nucleotide variants (SNVs), insertions and deletions (indels) in 55 genes, copy number amplifications (CNAs) in two [2] genes, and fusions in four [4] genes	FDA, Nov 2016 [122] to identify NSCLC patients who may benefit from treatment with the targeted therapies in accordance with the approved therapeutic products labelling
	FoundationOne Liquid CDx®	Targeted hybridization-based capture technology	cfDNA	311 genes panel including substitutions, insertions and deletions (indels), rearrangements and copy number losses only in BRCA1 and BRCA2	US FDA, August 2020 [149] to identify NSCLC patients who may benefit from treatment with the targeted therapies in accordance with the approved therapeutic products labelling

*FDA* Food and Drug Administration, *FFPE* Formalin-fixed paraffin-embedded

Patients with *EGFR* sensitising mutations treated with 1^st^ or 2^nd^ generation TKIs presented a profound overall response rate (ORR) around 60–70% but display a PFS of only 9 to 14 months [9, 10, 65]. The T790M mutation is the most frequent mechanism [12, 13] and is associated with response to 3rd generation TKIs [68], making detection crucial for selecting candidates for this treatment. In this context, re-biopsy is even more difficult, not succeeded in 20–30% of patients [69–72] due to inaccessible tumour localisation sites, patients’ fragility, or increased risk for tissue biopsies. Therefore, LB assumes a relevant role in progressive disease. The usefulness of plasma for detecting T790M mutation was addressed in studies exploring the activity of 3rd generation TKIs. As main findings, the plasma detection rate of the T790M ranged from 51 to 81%, with specificity ranging from 77 to 100% [73–75] (Table 2), and dPCR and NGS-based assays displayed a higher sensitivity over the Cobas test (Table 4) [60]. Besides, plasma identified T790M resistance mutations missed by tissue biopsy due to tumour heterogeneity or inadequate or unavailable tumour tissue [59].

Regarding the predictive value of finding a T790M mutation in plasma, the response rate was similar, whether the T790M was identified in the plasma or tumour (ORR: 63 vs 62%) [76], suggesting that in patients with a plasma T790M positive assay, tissue biopsy could be avoided. Considering the rate of false-negative results observed (30%), the negative plasma results justify further investigation [76]. Similar findings were stated with 3^rd^ generation TKIs used in the 1^st^ line [77]. The details of approved *EGFR* plasma detection assays [67, 78, 79] are shown in Table 5.

In patients treated with 3^rd^ generation TKIs, several secondary resistance mechanisms may occur. The role of plasma genomic profiling of ctDNA was well documented in the trial where osimertinib was studied in patients with T790M-positive NSCLC. Out of the 73 patients included, 49% had no detectable T790M at progression, and 15% acquired an *EGFR* secondary mutation in C797S/G. Amplifications of *MET, ERBB2*, and Phosphatidylinositol-4,5-Bisphosphate 3-Kinase Catalytic Subunit Alpha (*PIK3CA)* were detected in 19%, 5%, and 4% samples, respectively. Other mechanisms of acquired resistance included mutations in B-Raf Proto-Oncogene, Serine/Threonine Kinase (*BRAF)* (V600E, 4%), Kirsten Rat Sarcoma Viral Proto-Oncogene (*KRAS)* (1%) and *PIK3CA* (E545K; 1%), and oncogenic fusion mutations in fibroblast growth factor receptor 3 *(FGFR3),* ret-proto-oncogene *(RET)* and *NTRK* (4%) [75]. The resistance mechanisms after frontline osimertinib therapy in 91 patients were analysed through plasma NGS, and as expected, they did not lead to the emergence of T790M mutation. Instead, the most common acquired resistance mechanisms detected were *MET* amplification (15%), *EGFR* C797S mutation (7%) and *ERBB2*, *PIK3CA* and *RAS* mutations (2–7%) [80]. Circulating tumour DNA NGS-based genotyping demonstrated an expanding value, capturing the clonal heterogeneity manifested by various resistance mechanisms and overcoming the difficulty in carrying out re-biopsies at progression.

## Circulating cell-free tumour DNA for detection of ALK rearrangements and ALK resistance mutations

The detection of an *ALK* rearrangement can be done in a tissue sample by fluorescence in situ hybridisation (FISH), immunohistochemistry (IHC), retro-transcription polymerase chain reaction (RT-PCR), or integrated into a multiplex test by NGS [81]. *EML4*-*ALK* translocation is challenging to detect in cfDNA due to the different possible breakpoints and the number of base pairs involved larger than the typical cfDNA fragments. Options to look for gene translocations are to search for genomic breakpoint junctions or to analyse cell-free RNA[82]. Unlike the analysis of cfDNA to detect mutations, which has already been validated and implemented, plasma RNA analysis is not yet routinely used, despite its feasibility. Technically, RNA isolation and conservation difficulties exceed those in DNA [83], and the sensitivity of RT-PCR is low [84], limiting its use in clinical practice. Other methods under investigation for *ALK* translocation detection are the CTC and circulating tumour-associated platelets [85]. However, CTC analysis is challenging to implement due to demanding pre-analytical requirements and a lack of clinical validation. Also, RNA released from tumour cells can be transported by vehicles as exosomes to circulant platelets and be extracted from the platelets to be analysed, although this technique is still under investigation [86].

Concerning LB for ALK fusion detection, results are promising. Generally, sensitivity is not as high as for *EGFR*, but 100% specificity ensures a high predictive positive value. A PCR-based target sequencing showed low sensitivity, 50% with 100% specificity [87]. Instead, with amplicon-based technology, *ALK* rearrangement detection sensitivity was 78% and 100% for *ROS1* [88]. With capture-based next-generation sequencing, sensitivity ranged from 50 to 79% with 100% specificity [61, 89, 90]. The agreement between tissue and plasma NGS for *ALK* rearrangements was acceptable in different studies, varying from 79.2 to 100% (Table 6).

**Table 6:** Major studies focus on the cfDNA plasma detection of ALK fusions

Reference	Method of detection	Study type/ Sample size	Sensitivity/ PPA	Specificity/ NPA	Concordance/ OPA between tissue and liquid biopsy
Kunimasa et al. [87]	PCR-based target sequencing ALK intron19	N = 20	50%	100%	n.r
Mezquita et al. [88]	Amplicon-based (InVision™)	Retrospective N = 59/6	ALK 78%/ROS1 100%	n.r	86%
Schwartzberg et al. [61]	NGS HiSeq® 2500 (Illumina)	Prospective N = 115	50%	100%	96%
Cui S et al. [89]	Capture-based NGS	N = 39	54%	100%	n.r
Wang Ye et al. [90]	Capture-based NGS	N = 24	79%	100%	92%
Horn L et al. [92]	Hybrid-capture system NGS (Resolution Bioscience)	(Phase I/II multicohort eXalt2 trial) N = 76 (22 with paired pre-treatment tissue and plasma)	n.r	n.r	91%
Dagogo-Jack I et al. [91]	Hybrid-capture next-generation sequencing	Prospective N = 22 with ALK progressive disease	86% ALK fusions 50% ALK mutations	n.r	100% 100%

*PPA* Positive percent agreement, *NPA* Negative percent agreement, *OPA* overall percent agreement. *n.r.* not reported

Despite the good results with *ALK* inhibitors, resistance is inevitable, where mutations in the *ALK* gene are one of the resistance mechanisms. ALK mutations are diverse and differ depending on each *ALK* inhibitor. For example, L1196M often occurs after treatment with crizotinib, G1202R with ceritinib or alectinib, F1174C with ceritinib and I1171T/N/S with alectinib [25]. New generation *ALK* TKIs, like lorlatinib, ensartinib and entrectinib are potent inhibitors that showed promising results for most resistance mutations [24]. Most studies addressing the resistance to *ALK* inhibitors have used ctDNA analysis as the dominant tool for detecting mutations and dynamic surveillance [91–93]. Dagogo et al. used a 566 hybrid-capture gene assay to perform a longitudinal analysis of plasma specimens from 22 *ALK*-positive patients with acquired resistance to *ALK* TKIs. At the disease progression, an *ALK* fusion and *ALK* resistance mutations were detected in plasma in 86% and 50% of patients, respectively, with 100% agreement between tissue- and plasma-detected *ALK* fusions [91]. LB will be essential for selecting and sequencing *ALK* inhibitors, and in this context, the use of NGS platforms is an asset. As for *EGFR* progression, non-targeted mechanisms are harder to capture with an LB. *MET* amplifications can occur in about 15% of patients treated with new-generation TKIs and rarely histological transformation and Epithelial-Mesenchymal Transition [26, 27]. Thus, it is advisable to pursue a tissue biopsy whenever no resistance mechanism is found in the liquid assay.

The predictive value of cfDNA for selecting patients for *ALK* TKI treatment was proven in a prospective trial to use blood-based NGS testing to identify actionable genetic alterations and allocate patients to targeted or immunotherapy; among the *ALK* cohort, ORR was 87.4% with the studied *ALK* TKI [94]. There is enough evidence for treating patients with an *ALK* fusion detected on an LB, as supported by the IASCL in the Perspective of the International Society of Liquid Biopsy (ISLB) [95]. However, consistent data correlating plasma findings with clinical outcomes remain scarce, and the standardisation of the methodology is lacking; therefore, clinical application is fragile and more prospective trials are needed.

## Circulating cell-free tumour DNA for detection of other oncogenic alterations

Beyond *ALK* rearrangements, other fusion transcripts from the *ROS1*, *RET* or *NTRK* genes are considered for targeted treatment. For the detection of these alterations, NGS applied to circulating nucleic acids can be helpful. Still, due to the rarity of these subsets, available data is minimal.

*ROS1* was punctually detected in studies evaluating NGS for LC genotyping [88, 96], stating that NGS-based assays can detect fusions partners accurately. In the study of Mezquita et al. [97], 67% of *ALK* and *ROS1* fusions were detected in LB specimens at diagnosis with an amplicon-based assay. Dagogo-Jack et al. [98] found that the sensitivity of plasma genotyping for detecting *ROS1* fusions was 50% with hybrid-capture plasma NGS. However, in another study (NILE study), only two *ROS1* positive patients had paired plasma and tissue samples, and in both, the rearrangement was solely detected in tissue [99]. Data from patients with different drivers progressing on TKIs where the emergence of *ROS1* fusions was present, revealed that plasma genotyping allowed to detect the same spectrum of *ROS1* fusions and genetic alterations mediating resistance observed in tissue [91]. However, negative results must be interpreted cautiously due to the limited sensitivity and lack of robust data.

*RET* alterations occur in different cancers, including LC [100]. In a large study involving multiple advanced cancers types, using a hybrid-capture targeted 70-gene cfDNA test, *KIF5B-RET* fusion was dominant in NSCLC, and that non-*KIF5B-RET* fusion contributed to anti-*EGFR* resistance, highlighting the importance of knowing the specific gene partner [101]. RET gatekeeper mutations (e.g. *RET* V804M and *RET* S904F) can mediate resistance to multikinase inhibitors [102], and solvent front mutations (e.g. *RET* G810) were described as a mechanism of resistance to the new selective *RET* inhibitor, selpercatinib [103]. In the late case, analysis was performed in ctDNA and confirmed in tissue [103]. The European Society of Medical Oncology (ESMO), Translational Research and Precision Medicine Working Group (TR and PM WG) recommendations on the methods to detect *RET* fusions and mutations for NSCLC advise NGS, and if it is not available, FISH or RT-PCR. Also, consider performing a cell-free nucleic acid NGS broad panel for patients whose tissue is unavailable or exhausted. Tumour testing is still required if a *RET* alteration is not detected in a LB [104].

*NTRK 1, NTRK 2* and *NTRK 3* fusions encode *NTRK* fusion oncogenic proteins involved in multiple infantile and adult cancers and are biomarkers for the use of TRK small molecule inhibitors [105]. NTRK fusion gene can be detected by IHQ, FISH, RT-PCR, and both RNA-based and DNA-based NGS. NGS platforms should include all fusions variants, including *NTRK2* and 3 that present large intronic regions. Also, targeted-RNA platforms are helpful for this kind of detection. The ESMO TR and PM WG evaluated the available methods used to detect these tumour-agnostic alterations for daily practice and clinical research in different scenarios [106]. In the scenario of testing an unselected population where NTRK1/2/3 fusions are uncommon, as it is in LC, either frontline sequencing (preferentially RNA-sequencing) or screening by IHQ followed by sequencing of positive cases is advised [106]. *NTRK* fusions and resistance mutations detection in cfDNA is feasible [107], awaiting further experience.

LB was used to detect the *MET* exon 14 skipping mutation in a clinical trial, the phase II VISION study, in which 66 out of 99 patients who entered the study were included based on the detection in the plasma and derived the same benefit as those detected on tissue [108]. Thus, and considering that all these events are rare in LC, ideally, the detection should be part of a strategy that allows the simultaneous screening of multiple targetable alterations. The contribution of cfDNA genotyping in this strategy will be clarified below.

## Liquid biopsy cfDNA NGS for genotyping untreated advanced lung cancer

In LC patients with advanced disease, identifying potentially treatable tumour genomic changes is a key element. Considering the current target drugs availability and current evidence, ESMO recommends routine use of NGS on tumour samples in advanced NSCLC, including *ALK, BRAF, EGFR, ERBB2, KRAS, MET, NTRK, RET* and *ROS1* genes [109, 110]. The panel may need to be expanded depending on the clinical or investigational setting [110].

Undoubtedly the sequential analysis, gene by gene, is impractical in the real-world setting, indicating the need for multiplex sequencing [111]. NGS is based on the massive and parallel sequencing of millions of different DNA molecules, allowing the detection of several mutations in multiple genes [112]. Initially used in tumour samples, as the sensitivity improved, it became possible to be applied to LB, with new platforms able to detect tiny fractions of tumour DNA in circulation. Unlike conventional plasma genotyping techniques, such as the Cobas test, or digital PCR, which detect specific mutations of a given probe, NGS techniques have the potential to genotype tumours more comprehensively [113]. Generally, NGS techniques can be amplicon-based or hybrid-capture based, accounting for differences in test performance and range of alterations detected [113].

For LC genotyping, cfDNA test performance depends on the technology used, with overall sensitivity around 70–81% and very high specificity, as proved in different studies (Table 7).

**Table 7:** Studies focus on the cfDNA plasma NGS for genotyping of newly diagnosed NSCLC

Study	Method	Sample size	Sensitivity	Specificity	Concordance tissue/liquid biopsy %
Conraud et al. [114]	NGS amplicon-based (ion Torrent PGM)	N = 68	Del19: 55% Exon 18 = 100% All = 58%		68%
Thompson et al. [115]	NGS 70 genes Guardant360 panel Illumina Hi-Seq 2500	N = 102	84% (50 drivers, 12 resistance and 22 in additional genes)	NA	60% (79% for EGFR mutations)
Leighl et al. [99]	NGS Guardant360CDX	N = 282	80% for any guideline-recommended biomarker		For (EGFR, ALK, ROS1, BRAF) concordance was > 98.2%
Aggarwal et al. [120]	NGS Guardant360CDX	N = 323			90%
Li et al. [117]	NGS hybrid capture panel covering 37 lung cancer-related genes	N = 127	75% for de novo plasma detection of known oncogenic drivers	100%	NA
Fernandes et al. [46]	NGS amplicon-based	N = 115	81%	95%	76%
Papadopoulou et al. [150]	NGS amplicon-based	N = 121 (36 with matched plasma and tissue)	49% at least one mutation detected 89% sensitivity for the matched population		86%
Mack et al. [121]	NGS Guardant 360	N = 8388	Somatic alterations were detected in 86% of samples. Activating alterations in actionable oncogenes were identified in 48% of patients, *EGFR* (26.4%), *MET* (6.1%), and *BRAF* (2.8%) alterations and fusions (*ALK*, *RET*, and *ROS1*) in 2.3%		
Schrock et al. [119]	NGS hybrid capture panel covering 62 lung cancer-related genes	N = 1552	Genomic alterations were detected in 86% of samples. Most frequent were: (TP53) (59%), EGFR (25%), and KRAS (17%)		

The BioCAST/IFCT-1002 was a pilot trial from Conraud and colleagues where a technology based on multiplex PCR covering 12 specific genomic regions covering the most relevant genes was used. Test's sensitivity was 58% and specificity 87% having tumour samples as reference [114]. In the work of Thompson et al., NGS-based LB found genomic changes in 84% of patients, 50 considered "drivers", 12 resistance and 22 additional changes in genes for which there were experimental therapies or clinical trials [115]. In untreated NSCLC patients with no tissue sample available, plasma NGS detected clinically relevant molecular changes in 23% [116], being extremely useful in that context. Our group used a DNA amplicon-based assay in a cohort of 115 Portuguese treatment-naive patients with paired tissue samples, attaining 81.0% sensitivity, 95% specificity, 95% PPV, 84% NPV, 88% and 76% concordance [46].

To improve gene fusions detection, Papadopoulou et al. used an amplicon-based NGS combined panel for cfDNA and cfRNA for the initial molecular characterisation of 121 NSCLC patients. The panel included 12 genes frequently altered in NSCLC and fusions in *ALK*, *ROS1* and *RET* genes. At least one mutation was found in 49% of patients, including one *EML4-ALK* translocation. Among the 36 patients with tissue paired samples, concordance was high (77 to 83%). Using ultra-deep NGS technology and filtering the clonal hematopoietic somatic mutations, the detection of de novo known oncogenic drivers with a hybrid capture panel covering 37 LC-related genes led to a sensitivity of 75% with 100% specificity [117]. Plagnol et al. validated an enhanced tagged amplicon sequencing (eTAm-Seq™) technology to profile 36 genes commonly mutated in NSCLC for actionable genomic alterations in cell-free DNA, including point mutations, indels, amplifications and fusions. This assay allowed the detection of *ALK* and *ROS1* gene fusions and DNA amplifications in *ERBB2, FGFR1, MET* and *EGFR* with high sensitivity and specificity [118]. Also, a large assay for initial genomic profiling studied ctDNA from 1552 patients with NSCLC with a hybrid capture-based of 62 genes. At least one genomic alteration was detected in 86% of cases, among which 32% was a targetable alteration according to NCCN guidelines. Also, kinases fusions were detected in 5% of cases in *ALK, RET, ROS1*, *FGFR3*, platelet-derived growth factor receptor alpha gene (*PDGFRA*), and platelet-derived growth factor receptor beta gene (*PDGFRB*). Furthermore, exon 14 *MET* skipping mutation was present in 1.9% of cases [119].

The clinical relevance of integrating cfDNA genotyping in metastatic NSCLC clinical management has progressively been proven. Leigh et al. conducted a large prospective trial, Non-invasive versus Invasive Lung Evaluation (NILE) [99], to demonstrate that a comprehensive cfDNA test used at diagnosis of metastatic NSCLC is non-inferior to that of physician discretion standard of care tissue genotyping to identify guideline-recommended genomic biomarkers. The authors found 80% cfDNA sensitivity for any guideline-recommended biomarker, including *EGFR* mutations, *ALK* fusions, *ROS1* fusions, *BRAF V600E* mutation, *RET* fusions, *MET* amplification and *MET* exon 14 skipping variants, and *ERBB2* mutations. For FDA-approved targets (*EGFR, ALK, ROS1, BRAF*), the concordance was 98.2%, with 100% positive predictive value for cfDNA versus tissue. Also, when using cfDNA in addition to tissue, the detection increased by 48%, including in patients with negative, not assessed, or insufficient tissue results. The median turn-around time for cfDNA was significantly faster than that of tissue (9 vs 15 days; P < 0.0001) [99]. In another crucial study, from a “real-world” clinical setting, Aggarwal et al. demonstrated that the integration of plasma NGS testing into the routine management of stage IV NSCLC increased the detection of therapeutically targetable mutations [120]. Recently, in one of the most extensive studies with ctDNA on 8388 advanced NSCLC patients, activating alterations in actionable oncogenes were identified in 48% of patients, including *EGFR* (26.4%), *MET* (6.1%), and *BRAF* (2.8%) alterations and fusions (*ALK*, *RET*, and *ROS1*) in 2.3% [121].

These studies confirm that a cfDNA comprehensive analysis is powerful to detect targetable genomic alterations in untreated NSCLC patients. At present, blood-based genomic profiling-based clinical trials are being conducted. More results from the BFAST, phase II/III global, multi-cohort study evaluating blood-based NGS detection of actionable genetic alterations in ctDNA for selecting patients for 1^st^ line targeted therapies/immunotherapy will elucidate the predictive value of LB, as already stated for *ALK* [94]. Both the Guardant360 CDx® assay (Guardant Health, Redwood City, CA) [122] and the Foundation One Liquid CDx® test (Foundation Medicine, Inc.) are approved for multiple biomarkers detection in cfDNA isolated from plasma specimens [123] (Table 5). Other platforms are under investigation and approval process.

## Clinical value of liquid biopsy for monitoring treatment response and progression

Currently, tumour response evaluation is based on radiology RECIST criteria [124] and complemented with functional images. This evaluation represents an isolated timepoint, dependent on the exam resolution and exposing patients to radiation. At progression, tumours had been suffering from temporal and therapeutic selective pressure and cancer heterogeneity [31], and clonal divergence from the primary tumour emerges as an obstacle to be overcome and thus needs to be considered in subsequent therapeutic options [125]. As a potential representative of all shedding tumour focus with each clonal expression, LB is a potential tool to face this challenge. Molecular disease monitoring has three significant purposes: monitoring disease burden as an indicator of tumour response or relapse, monitoring clonal evolution by analysing variations of the variant allelic fractions and detecting the emergence of resistance mechanisms.

One of the first studies approaching response through LB was the FASTACT-2 trial, chemotherapy interspersed with erlotinib. Blood persistence of *EGFR* mutation after an 8-week treatment was linked to a poor prognosis [125]. The PFS was lower in patients who maintained detectable levels of *EGFR* mutation in plasma after 2-month treatment, 6.3 vs 10.1 months [126]. Also, as with 1^st^ line *EGFR* TKI treatment, early disappearance, within 6 weeks, of the T790M mutation was associated with better clinical outcomes with osimertinib treatment [127]. Serial ctDNA analysis can detect the appearance of T790M before radiological progression defined by the RECIST criteria. Zheng and colleagues detected the T790M mutation at the median of 2.2 months before radiological progression [57]. The mutation was present before radiological progression in another series as early as 344 days [128]. Likewise, after 3rd generation TKI treatment, changes in plasma T790M levels were detected, in most cases mirroring the clinical and radiological evolution [129]. Among our patients submitted to ctDNA longitudinal monitoring, a decrease in variant allelic frequency (VAF) or clearance of mutant alleles was associated with response, while an increase or emergence of novel alterations was linked to progression. In most cases, such variations anticipated radiographic changes, with a median time of 0.86 months [47].

Clonal monitoring has been integrated into recent trials involving new generation *ALK* inhibitors. For example, Dagogo et al. demonstrated with serial plasma sampling that *ALK* mutations emerged and disappeared during treatment with sequential *ALK* TKIs, and that such data was helpful to guide TKIs selection [91]. Also, Shaw et al. studied the efficacy of lorlatinib among patients with and without *ALK* mutations using plasma or tissue genotyping [130]. For plasma genotyping, PFS did not differ significantly in patients with and without *ALK* mutations [130], meaning that plasma negative patients include true negative cases and some false negatives that are positive on tissue. Therefore, like in the *EGFR* T790M context, tissue confirmation must be pursued whenever possible in case of a negative plasma result.

These findings reinforce the need to monitor the disease in a model that integrates clinical progression assessed by symptoms, radiological (RECIST) and clonal finding through monitoring the ctDNA. Nevertheless, from a clinical point of view, the most pertinent question is whether early detection, prior to radiological, and the consequent anticipation of therapeutic change will translate into more favourable clinical outcomes. To date, there is no data available supporting this hypothesis. The results from The AZD9291 (Osimertinib) Treatment on Positive Plasma T790M in EGFR-mutant NSCLC Patients (APPLE Trial) as well as of similar studies are expected to confirm the value of LB for the decision-making process [131].

## Liquid biopsies pitfalls

Considering the increasing accuracy and the conquered role in guiding clinical decisions, adopting LB in LC management, specifically cfDNA analysis, is inevitable. Still, it is indispensable to understand the LB limitations and drawbacks. First of all, there is some discrepancy between cfDNA results and paired tissue samples relating to the reduced sensitivity of cfDNA responsible for false negatives. Cell-free DNA analysis is technically demanding, requiring rigorous standardised protocols for plasma collection preservation, DNA isolation, library preparation and sequencing, being susceptible to failures in those multiple pre-analytical steps [41, 45, 132]. Regarding sequencing analytics, understanding the accuracy of the test and the range of hotspots covered is essential and is a new requirement for the clinician. As an example, not all assays can detect gene amplifications and rearrangements, requiring appropriate technologies, as elucidated before. Also, accurate post-analytical procedures are needed to avoid misinterpretations. In this sense, expertise in bioinformatics is paramount in interpreting findings, distinguishing germline alterations and clonal haematopoiesis-related alterations from oncogenic tumour mutations, and avoiding false-positive results [42, 45, 132]. Detected variants must be reported according to the Association for Molecular Pathology (AMP), American Society of Clinical Oncology (ASCO), and College of American Pathologists [133].

The intrinsic nature of the disease can compromise LB results. Some tumours release low or no DNA to the circulation (*non-shedders*) [134]. The amount of ctDNA is related to the disease stage, tumour burden, localisation, and size of the metastasis, particularly limited in less extensive, oligometastatic disease and exclusively brain metastization [46, 135, 136]. Also, cfDNA profiling does not allow the morphologic characterisation of the tumour, PDL1 IHQ assessment, and rule out histological transformation.

Thus, considering the clinical context and the exposed limitations, a proper interpretation requires collaborative efforts between clinicians, pathologists and molecular biologists gathered in a Mutational Tumour Board to optimise treatment personalisation and contribute to accurate precision medicine.

## Interpretation of liquid biopsy results

The predictive value of ctDNA findings supports LB reliability for clinical decisions. As exposed above, ctDNA genotyping revealed extremely high analytic specificity and positive predictive value, making false positives improbable. In addition, identifying oncogenic mutations through the ctDNA analysis predicts the clinical response in a similar magnitude of tumour detection. Therefore, if a mutation is detected, it is probably a true positive result and identifies candidates for treatment. However, due to the low sensitivity, a negative test does not exclude the presence of a mutation and results must be designated as uninformative or alteration(s) not detected. It is advised confirmation through tissue biopsy.

On the opposite, but less frequent, is detecting oncogenic alterations in plasma not present in the tissue, which can be considered a false positive if tissue is the reference. This can occur due to tumour heterogeneity, with some alterations not being expressed in the correspondent sample, especially concerning progression, where “de novo” alterations are expected to occur. It is not a handicap of LB but an advantageous, expressing the complementary role to tissue analysis. Genuine false positives are rare and can be associated with analytic or interpretation errors, different tumour origin or clonal haematopoiesis [95]. In the last case, germline cell sequencing can help exclude this.

**Fig. 1:**
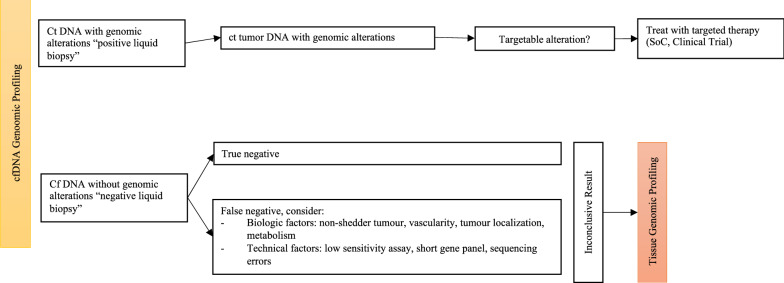
Proposed algorithm for clinical interpretation of a liquid biopsy for the detection of targetable mutations. For clinical interpretation, a targetable alteration found in a liquid biopsy is considered a true positive finding and is used to guide treatment selection. A liquid biopsy with no detectable alteration must be confirmed with a tissue biopsy to overcome false negative results

Considering the *EGFR* mutated scenario as the paradigmatic example, LB is the first test to look for the T790M mutation, as recommended [4]. If the resistance mutation is found in plasma (positive test), the patient is eligible for treatment with a 3^rd^ generation inhibitor. On the other hand, a tissue biopsy is advised when the T790M mutation is not detected. The absence of plasma mutation (negative test) may occur because the resistance mechanism is another, due to a false-negative attributed to the test's low sensitivity or the absence of "secretion" for the circulation of DNA through the tumour. In the latter case, the initial driver mutation will also not be present. In cases where it is not possible to perform a tissue biopsy, the LB can be repeated, and as the tumour or its metastases growths, it may "release" more ctDNA, allowing to detect the T790M. Depending on the context, other alternatives to plasma ctDNA may be other biological fluids, like CSF [136]. The same rationale for interpretation applies to the other oncogenic alterations found with a plasma assay, as illustrated in Fig. 1.

## Future perspectives

LB conquered a definitive place in the management of patients with advanced or metastatic LC. Future perspectives will embrace expanding its application to immunotherapy and less advanced stages of the disease.

Concerning NSCLC advanced disease, targeted treatments produce remarkably high and sustained response rates, contributing to the incremental survival observed in the last decade in NSCLC patients [137] and must be the first treatment option. The following options are immunotherapy with checkpoint inhibitors alone or combined with chemotherapy [3, 4]. The only validated predictive factor for selecting patients for immune checkpoint inhibition is tissue expression of PD-L1[4], which has numerous limitations that are beyond the scope of this review. Other biomarkers have been explored, namely Tumour Mutation Burden (TMB)[138]. Briefly, TMB refers to the number of nonsynonymous mutations per megabase. Hypothetically, a high TMB correlates with patients' responses to treatment with PD-1/PD-L1 inhibitors [130]. Blood TMB (bTMB) has been investigated in clinical trials with different plasma-based NGS platforms. For example, in patients treated with atezolizumab, a high bTMB (> 16 SNVs, detected among 394 genes) correlated with the response with the FoundationOne CDx NGS assay [139], pointing to blood TMB as a surrogate of tissue TMB. However, there is controversy regarding concordance between tissue TMB and cfDNA TMB, particularly when different assays are compared [140]. Therefore, adopting blood TMB requires additional validation and harmonisation of the technical aspects [140]. In addition, other specific mutations, such as *KRAS, TP53, STK11* and *PTEN* have been described as influencing the response to immune checkpoint inhibitors and can be detected and tracked in the blood [141, 142]. Future perspectives shall explore the role of LB in patient selection, response evaluation, disease monitoring and interpretation of pseudo-progressive disease. Clinical trials embracing genomics with immunotherapy must be held.

Moving to the role of LB in localized disease, the detection and molecular characterisation of minimal residual disease (MRD) is of particular importance. MRD evaluation can improve patient selection for adjuvant therapy, contributing to clinical outcomes while avoiding overtreatments [44]. Several studies have suggested that ctDNA can be used to detect the presence of MRD after surgical resection in several cancer types, including LC, by documenting a marked decline in presurgical and postsurgical levels of ctDNA [143, 144]. Additional data in support of using ctDNA-based MRD detection was obtained from the TRACERx trial [145]. This study created an individualized panel of single-nucleotide variants for each patient using exome sequencing of their primary tumour. The results demonstrated that ctDNA status was closely linked to disease relapse after intent-to-cure surgery [145]. Subsequently, another study reported the application of CAPP-Seq to assess for MRD. Detectable ctDNA was found in 72% of all patients who exhibited radiographic progression and preceded these findings by a median of 5.2 months. The results of these studies together imply a robust potential role of ctDNA-assessed MRD [146] that must be further explored. Promising results of the application of cfDNA to early cancer detection are ongoing and technical advances are expected to overcome the sensitivity and specificity limitations inherent to the study of an asymptomatic and low burden disease population. Furthermore, ctDNA epigenetic markers in plasma can be detected early during cancer pathogenesis and provide information on early detection, prognosis, MRD, and therapy response and will open a new era in the LB field [147]. Finally, incorporating ctDNA in clinical trial design in the different scenarios of LC management is becoming indispensable and must be accomplished.

## Integrating cfDNA comprehensive genomic tumour profile in lung cancer management

**Fig. 2:**
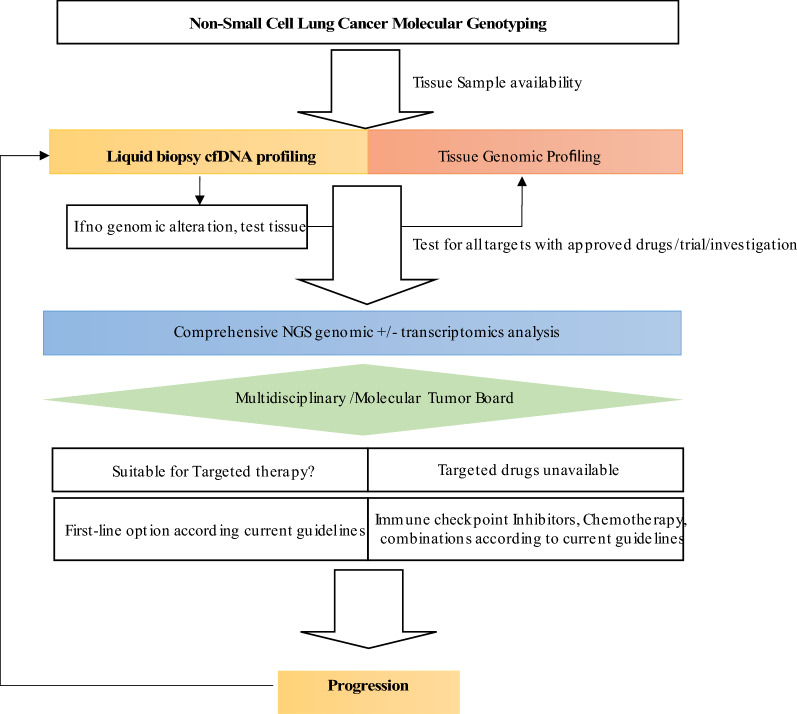
Proposed workflow integrating liquid biopsy in the management of advanced NSCLC

Integrating a comprehensive genomic tumour profile will be the cornerstone for LC management, and cfDNA will be an indispensable tool, as proposed in Fig. **2**. Circulating-tumour DNA genotyping is, at least, complementary to tissue genotyping, with the potential of having a better cost/efficacy profile with a shorter turn-around time [99]. Head-to-head comparison of a liquid-first *versus* tissue-first genotyping strategy, using the same NGS platform, with a comprehensive analysis of costs and associated health resources expenditure is eagerly needed. For detection of resistance mechanisms, evidence corroborates LB as the first step test, with tissue biopsy as a backup for negative results.

## Conclusion

The therapeutic decision in advanced LC stages is complex, involving several parameters. Clinical and functional evaluation of the patient condition and disease extension, combined with tumour morphological, immunohistochemical and molecular characterization, is paramount for clinical decision. As the disease progresses, all those factors are susceptible to changes, including the emergence of resistance mechanisms. Through ctDNA genomic profiling, a LB will more likely be the choice to identify genomic alterations in untreated patients and monitor and detect resistance mechanisms, as it embraces tumour heterogeneity, is non-invasive and repeatable. The LB will have a promissory impact on LC patients' survival and quality of life.

## Data Availability

Not applicable.
